# Safety and Effectiveness of Probiotic Preparations: A Contemporary Review

**DOI:** 10.3390/diseases14090316

**Published:** 2026-08-29

**Authors:** Erik Shorabaev, Amankeldi Sadanov, Baiken Baimakhanova, Irina Ratnikova, Gulzakira Xetayeva, Sholpan Akhelova, Aknur Turgumbayeva

**Affiliations:** 1 Research and Production Center for Microbiology and Virology LLP, 105 Bogenbay Batyr St., Almaty 050010, Kazakhstan; eshorabaev@mail.ru (E.S.); a.sadanov1951@gmail.com (A.S.); bbbayken@mail.ru (B.B.); iratnikova@list.ru (I.R.); 2 Department of Pediatric Infectious Diseases, S.D. Asfendiyarov Kazakh National Medical University, 94 Tole Bi St., Almaty 050012, Kazakhstan; 3 Department of Pharmaceutical Disciplines, Astana Medical University, 33 Saryarka Ave., Astana 010000, Kazakhstan; 4 Faculty of Medicine and Health Care, Farabi University, 71 Al-Farabi Ave., Almaty 050040, Kazakhstan; turgumbayeva.aknur@med-kaznu.com

**Keywords:** probiotics, gut microbiota, probiotic preparations, intestinal barrier, antibiotic-associated diarrhea, *Clostridioides difficile* infection, inflammatory bowel disease, irritable bowel syndrome, probiotic safety

## Abstract

Background/Objectives: Probiotic preparations have attracted increasing attention because of their potential to modulate the gut microbiota and improve health outcomes in a wide range of gastrointestinal and extraintestinal diseases. However, their efficacy and safety are strain-specific and remain inconsistent across many clinical indications. The aim of this review was to evaluate current evidence regarding the efficacy, safety, mechanisms of action, and clinical applications of probiotic preparations. Methods: A narrative literature review was conducted using the PubMed, Scopus, and Web of Science databases to identify publications addressing the efficacy, safety, and clinical applications of probiotic preparations. The literature search was last updated in July 2026. No publication-year restrictions were applied; the studies included in this review were published between 2000 and 2026. Priority was given to systematic reviews, meta-analyses, randomized controlled trials, international clinical practice guidelines, and mechanistic studies. Results: The reviewed evidence indicates that probiotics may contribute to intestinal homeostasis through modulation of the gut microbiota, enhancement of intestinal barrier function, and regulation of immune responses. The strongest evidence supports the use of selected probiotic strains for the prevention of antibiotic-associated diarrhea, whereas evidence for the prevention of necrotizing enterocolitis is generally favorable but depends on the specific probiotic preparation, product quality, and clinical setting. Evidence for most other gastrointestinal and extraintestinal disorders remains heterogeneous and strain-specific. Although probiotics generally exhibit a favorable safety profile, rare infectious complications have been reported in high-risk patients. Conclusions: Current evidence indicates that the efficacy and safety of probiotics depend on the specific strain, clinical indication, and patient characteristics. Further high-quality, strain-specific clinical studies are needed to optimize their use in medical practice.

## 1. Introduction

The rapid expansion of research on the gut microbiota has led to growing interest in probiotic preparations as promising tools for the prevention and treatment of a wide range of diseases [1,2,3]. This interest is largely attributed to their ability to exert beneficial effects on human health through modulation of the composition and function of the gut microbiota [3,4]. The beneficial effects of probiotics are mediated by their capacity to maintain microbial homeostasis, inhibit the growth of pathogenic microorganisms, reduce intestinal pH, and modulate host immune responses [5]. The growing importance of this field is reflected in the substantial increase in scientific output: the number of publications on probiotics and their role in human health and disease has increased more than 30-fold since the late twentieth century, and international scientific databases now contain thousands of studies devoted to this topic [6].

In recent years, increasing attention has been devoted to the role of the gut microbiota as one of the key determinants of human health [7,8]. The gut microbiome is a complex ecosystem comprising trillions of microorganisms that contribute to the regulation of digestion, metabolism, the synthesis of vitamins and other bioactive compounds, and the development and function of the immune system [7,9]. In addition, the gut microbiota plays an essential role in maintaining intestinal barrier integrity and protecting the host against colonization by pathogenic microorganisms [10]. Disruption of these functions resulting from alterations in the composition and activity of the gut microbiota, commonly referred to as dysbiosis, is considered an important contributor to the development of a wide range of diseases. Growing evidence indicates that changes in gut microbial composition can influence multiple physiological systems and contribute to the pathogenesis of both gastrointestinal and extraintestinal disorders [9,11,12].

Although the term *probiotic* was introduced relatively recently, the consumption of fermented foods with potential probiotic properties has a history spanning several millennia. Products such as kefir, koumiss, yogurt, and various types of cheese have been traditionally consumed long before the discovery of microorganisms and the recognition of their role in human health [13]. A major milestone in the development of the probiotic concept was the adoption of the official definition by the expert consultation of the Food and Agriculture Organization of the United Nations (FAO) and the World Health Organization (WHO) in 2001, which defined probiotics as “live microorganisms which, when administered in adequate amounts, confer a health benefit on the host” [14,15].

Over the past decades, advances in our understanding of the gut microbiota have led to the development of next-generation probiotics and live biotherapeutic products with potential therapeutic applications [16]. Currently, probiotics are regarded as a promising approach for the prevention and management of gastrointestinal diseases, including gastrointestinal infections, irritable bowel syndrome, inflammatory bowel diseases, and antibiotic-associated diarrhea, as well as selected extraintestinal disorders such as allergic, metabolic, cardiovascular, and neuropsychiatric diseases [5,7,12,17,18]. Among the most consistently supported clinical applications of probiotics is the prevention of antibiotic-associated diarrhea [17]. However, for many other clinical indications, the available evidence remains inconsistent, highlighting the need for further well-designed studies.

The principal mechanisms of probiotic action include restoration of microbial balance, reinforcement of intestinal barrier function, modulation of host immune responses, and inhibition of pathogenic microorganisms through competitive interactions and the production of antimicrobial substances [3,19]. However, the clinical efficacy of probiotics is not universal and largely depends on the specific microbial strain and the disease being treated [20]. An additional challenge is the considerable variability among probiotic preparations with respect to strain composition, the concentration of viable microorganisms, and manufacturing quality, which complicates comparisons across clinical studies [21,22]. Furthermore, although most studies indicate that probiotics have a favorable safety profile, the available evidence remains insufficient to fully assess the risk of rare adverse events and the long-term consequences of probiotic use [23].

Accordingly, the aim of the present review is to summarize the current evidence regarding the efficacy and safety of probiotic preparations, critically evaluate their mechanisms of action and clinical applications across a range of diseases, and discuss future perspectives for their use in medical practice.

## 2. Methods

A narrative literature review was conducted using the PubMed, Scopus, and Web of Science databases to identify relevant studies evaluating the efficacy, safety, mechanisms of action, and clinical applications of probiotics. Particular attention was given to gastrointestinal and selected extraintestinal diseases, safety, quality, delivery technologies, and future therapeutic perspectives.

The search was last updated in July 2026. No restrictions on publication year were applied; however, studies published between 2000 and 2026 were considered based on their relevance to the scope and objectives of the study. The identified evidence was analyzed and thematically synthesized.

The search strategy combined Medical Subject Headings (MeSH), where applicable, with free-text keywords using the Boolean operators AND and OR. The principal search terms included “probiotics”, “probiotic preparations”, “gut microbiota”, “intestinal barrier”, “antibiotic-associated diarrhea”, “*Clostridioides difficile* infection”, “irritable bowel syndrome”, “inflammatory bowel disease”, “ulcerative colitis”, “Crohn’s disease”, “acute infectious diarrhea”, “necrotizing enterocolitis”, “infantile colic”, “atopic dermatitis”, “psychobiotics”, “obesity”, “metabolic syndrome”, “safety”, “adverse events”, “bacteremia”, “fungemia”, “antibiotic resistance”, “quality control”, and “encapsulation”. Additional combinations of disease-specific, strain-specific, and mechanistic keywords were used to ensure comprehensive coverage of the available literature.

An example of the primary PubMed search strategy was as follows: (“Probiotics”[Mesh] OR probiotic*[tiab]) AND (“antibiotic-associated diarrhea”[tiab] OR “*Clostridioides difficile*”[tiab] OR “irritable bowel syndrome”[tiab] OR “inflammatory bowel disease”[tiab] OR “Crohn disease”[tiab] OR “ulcerative colitis”[tiab] OR “necrotizing enterocolitis”[tiab]). Equivalent search strategies adapted to the indexing systems of Scopus and Web of Science were also applied.

Retrieved publications were assessed for relevance to the scope and objectives of this review. Titles and abstracts were examined to identify literature addressing the predefined thematic areas, and full texts were consulted where necessary to determine their relevance and scientific contribution to the narrative synthesis. Duplicate records, conference abstracts, editorials, letters to the editor, and publications outside the scope of the review were excluded. Reference lists of relevant reviews and selected articles were also manually examined to identify additional pertinent publications. Literature selection was guided by relevance and scientific contribution rather than by a predefined systematic review protocol. Accordingly, the evidence was synthesized qualitatively within a narrative framework.

**Figure 1:**
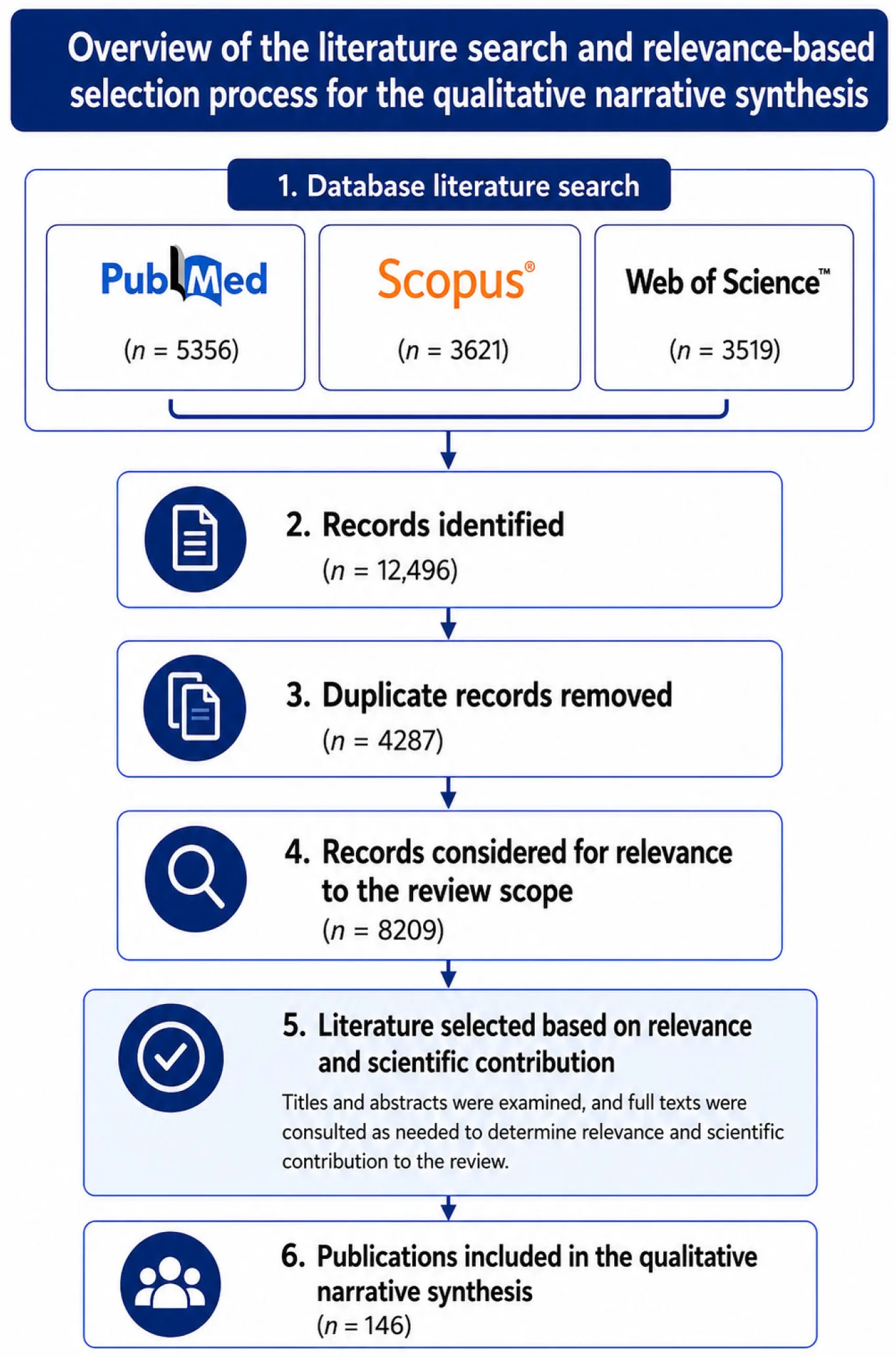
Overview of the literature search and relevance-based selection process used for the qualitative narrative synthesis.

This review included original research articles, randomized controlled trials, observational studies, systematic reviews, meta-analyses, narrative reviews, clinical practice guidelines, consensus statements, and other peer-reviewed publications relevant to the objectives of this review. Priority was given to higher levels of evidence, including systematic reviews, meta-analyses, randomized controlled trials, and international clinical practice guidelines. Experimental in vitro and animal studies were included when they provided mechanistic insights relevant to the biological effects of probiotics or helped interpret clinical findings. An overview of the literature search and relevance-based selection process is presented in Figure 1.

## 3. Probiotics: Definition, Classification, and Biological Characteristics

The use of fermented foods containing microorganisms with potential probiotic properties dates back centuries. Significant contributions to the development of the probiotic concept were made by the studies of Louis Pasteur, as well as the pioneering work of Élie Metchnikoff and Henri Tissier, who were among the first to associate specific groups of microorganisms with potential health benefits [17]. These discoveries laid the foundation for subsequent research into microorganisms capable of exerting beneficial effects on the host. The term *probiotic* was first introduced in 1965; however, its current internationally accepted definition was established much later [22]. In 2001, an expert consultation convened by the FAO and supported by the WHO proposed the first internationally accepted definition of probiotics [14,17,22,24,25]. In 2002, guidelines for the interpretation and practical application of this definition were published. Subsequently, in 2014, the International Scientific Association for Probiotics and Prebiotics (ISAPP) reaffirmed the FAO/WHO definition, which remains the internationally accepted standard in the scientific literature [14,22]. According to current international recommendations, a microorganism can only be considered a probiotic if its identity has been established at the strain level and its beneficial health effects have been demonstrated in studies conducted in the target population [26].

Probiotic microorganisms comprise diverse groups of bacteria and yeasts that are capable of exerting beneficial effects on host health. Among bacterial probiotics, members of the genera *Lactobacillus*, *Bifidobacterium*, and *Bacillus* are the most widely used, together with selected strains of *Enterococcus* spp. and *Streptococcus thermophilus*. Among yeast probiotics, *Saccharomyces boulardii* is the most extensively studied and widely used species [27]. Following the taxonomic reclassification of the former genus *Lactobacillus* in 2020, many species were assigned to newly established genera. Throughout this review, the updated taxonomic nomenclature is used where appropriate.

Members of the genus *Lactobacillus* are Gram-positive, non-spore-forming rod-shaped bacteria that constitute an important component of the normal human microbiota. They primarily inhabit the oral cavity, gastrointestinal tract, and urogenital tract, where they contribute to digestion, nutrient metabolism, and protection against pathogenic microorganisms. Owing to their ability to produce lactic acid and their well-established safety profile, *Lactobacillus* species are among the most widely used probiotic microorganisms [28,29]. In addition, lactobacilli are the dominant members of the vaginal microbiota in healthy women, where they help maintain an acidic environment and prevent colonization by pathogenic microorganisms [30]. The best-known representatives include *Lactobacillus acidophilus*, *Lacticaseibacillus casei*, *Lactiplantibacillus plantarum*, *Limosilactobacillus reuteri*, and *Lactobacillus delbrueckii* subsp. *bulgaricus*, all of which are widely incorporated into probiotic preparations and functional foods [24].

Members of the genus *Bifidobacterium* are important constituents of the normal human gut microbiota and are among the most extensively studied probiotic microorganisms. They play a key role in maintaining intestinal mucosal homeostasis, strengthening intestinal barrier function, and regulating host immune responses. In addition, bifidobacteria are among the earliest colonizers of the human gastrointestinal tract and are considered key members of a healthy gut microbiota, contributing to the maintenance of microbial homeostasis [31]. They also ferment carbohydrates to produce short-chain fatty acids, including acetate and propionate, which contribute to host metabolic and immune homeostasis [32]. Studies have shown that the abundance of bifidobacteria in the gut microbiota declines with age, reflecting age-related changes in microbial composition. The species most commonly used in probiotic preparations include *Bifidobacterium bifidum*, *Bifidobacterium breve*, and *Bifidobacterium animalis* [33].

Members of the genus *Saccharomyces* are yeast microorganisms used as probiotics. The best-studied and most widely used representative is *Saccharomyces boulardii*, a probiotic yeast taxonomically classified within the species *Saccharomyces cerevisiae*, which is used for the prevention and treatment of various forms of diarrhea and certain gastrointestinal disorders. Unlike many bacterial probiotics, *S. boulardii* is not a permanent member of the human gut microbiota and exerts its probiotic effects during transit through the gastrointestinal tract. Owing to its intrinsic resistance to antibacterial agents, *S. boulardii* can be administered concomitantly with antibiotic therapy and is widely used for the prevention of antibiotic-associated diarrhea [34].

Members of the genus *Bacillus* are spore-forming bacteria that are widely used in probiotic preparations [35,36]. Owing to their ability to form highly resistant spores, they exhibit remarkable tolerance to adverse environmental conditions and maintain their viability during passage through the gastrointestinal tract, as well as throughout the manufacturing, storage, and transportation of probiotic products [35,36,37]. In addition, *Bacillus* species produce antimicrobial compounds and are considered promising probiotic microorganisms because of their combination of environmental resilience and probiotic activity [37]. Studies have demonstrated that *Bacillus* spores are capable of germinating and transiently colonizing the host gastrointestinal tract, thereby exerting beneficial effects on the composition and function of the gut microbiota. The species most commonly used as probiotics include *Bacillus subtilis*, *Bacillus clausii*, *Bacillus coagulans*, *Bacillus pumilus*, and *Bacillus licheniformis* [36].

Other bacterial probiotics include selected strains of the genera *Enterococcus* and *Streptococcus*. *Enterococcus* strains are used in probiotic preparations because of their ability to maintain microbial balance in the gut and inhibit the growth of certain pathogenic microorganisms. However, their use as probiotics requires careful safety assessment because of the potential risk of horizontal transfer of antibiotic resistance genes [38]. In addition, certain *Enterococcus* strains produce bacteriocins (enterocins) with antimicrobial activity against antibiotic-resistant bacteria [39]. *Streptococcus thermophilus* is a lactic acid bacterium with a long history of safe use in fermented dairy products [40]. It participates in lactose fermentation, producing lactic acid, and exhibits probiotic potential by improving lactose tolerance and supporting gastrointestinal health [40,41]. Furthermore, certain strains of *S. thermophilus* have been shown to enhance intestinal mucosal barrier function and modulate local immune responses [42].

Based on their composition, probiotics are classified as single-strain or multi-strain formulations, containing one or multiple probiotic strains, respectively. Although synergistic interactions among microorganisms within multi-strain formulations have been proposed, their clinical efficacy is primarily determined by the properties of the individual strains and does not necessarily exceed that of single-strain probiotics [43,44]. A separate category comprises synbiotics, which combine probiotics and prebiotics to achieve complementary effects [45,46,47]. Synbiotics are intended to provide complementary or potentially synergistic effects through the combined action of probiotics and prebiotics. However, their superiority over individual probiotic or prebiotic preparations has not been consistently demonstrated across clinical indications [45,46]. To improve microbial stability and viability, encapsulation technologies have also been developed to protect probiotic cells and enhance their delivery to the gastrointestinal tract [48]. In addition to pharmaceutical preparations, probiotics are widely incorporated into functional foods, including fermented dairy products, beverages, and other food matrices, thereby facilitating the regular consumption of beneficial microorganisms and expanding their potential for disease prevention [24,49,50].

**Figure 2:**
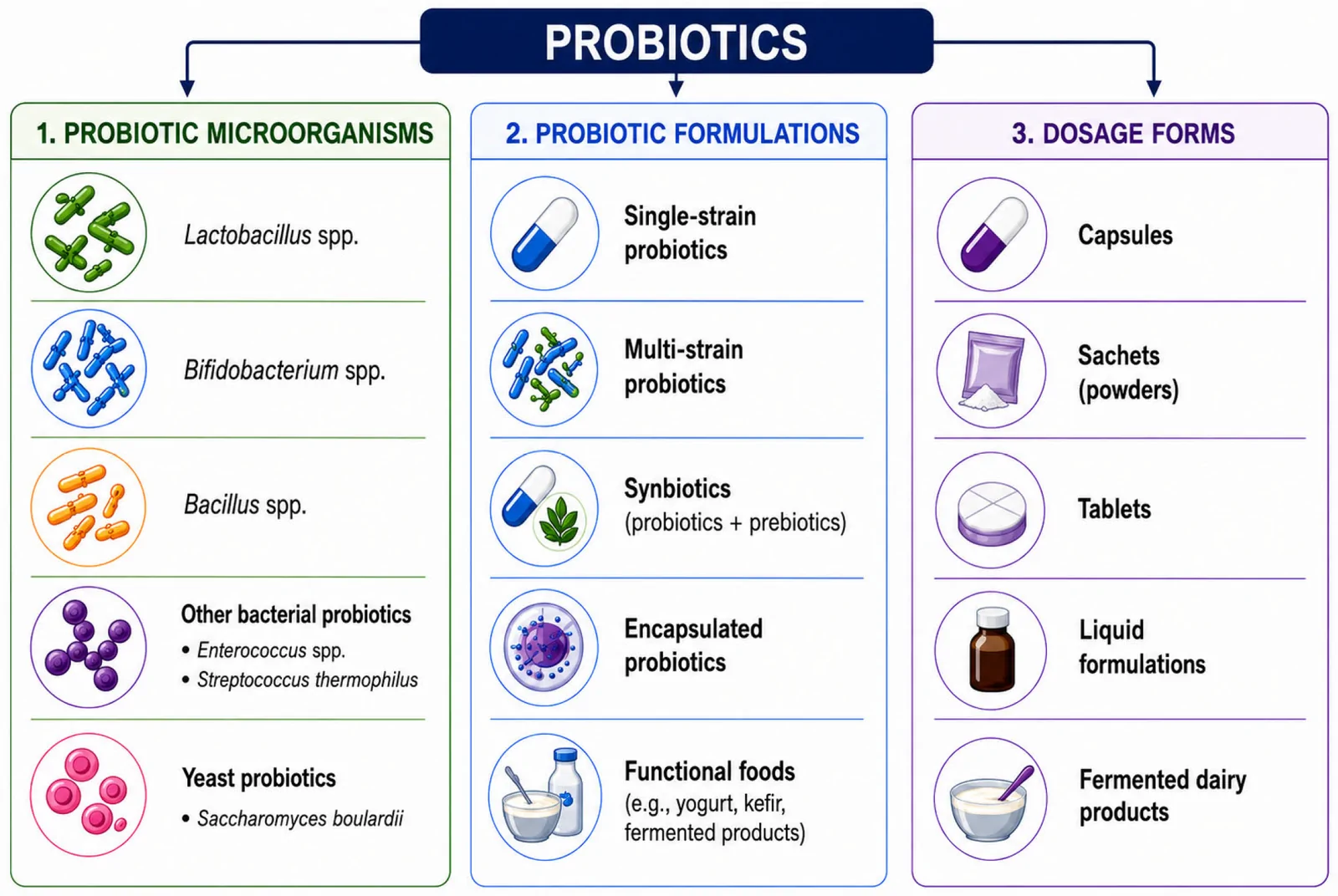
Schematic classification of probiotic microorganisms, formulations, and dosage forms. Probiotic microorganisms include bacterial and yeast species. Probiotic formulations comprise single-strain probiotics, multi-strain probiotics, synbiotics, encapsulated probiotics, and functional foods, whereas the principal dosage forms include capsules, sachets, tablets, liquid formulations, and fermented dairy products.

Probiotics are available in a variety of dosage forms designed to facilitate their use in different patient populations. The most common formulations include capsules, tablets, powders, sachets for oral suspension, and liquid preparations containing live probiotic cultures. The choice of dosage form depends on the characteristics of the probiotic strain, storage requirements, route of administration, and the intended target population [19,21,51]. The major groups of probiotic microorganisms, common probiotic formulations, and dosage forms are summarized in Figure 2.

## 4. Technologies for Probiotic Delivery, Protection, and Encapsulation

Probiotic microorganisms are highly sensitive to various environmental factors and conditions encountered within the gastrointestinal tract, which may reduce their viability and clinical effectiveness. Following oral administration, they are exposed to gastric acidity, digestive enzymes, and bile salts, while during manufacturing and storage they are subjected to temperature fluctuations, oxidative stress, and osmotic stress [51]. Therefore, technologies for probiotic delivery and protection have become increasingly important for preserving microbial viability and functional activity.

The drying process has a substantial impact on probiotic viability, cell surface hydrophobicity, resistance to environmental stress, and antimicrobial activity. Several dehydration techniques are used for probiotic cultures, including freeze-drying (lyophilization), spray-drying, vacuum drying, and fluidized-bed drying, although freeze-drying and spray-drying remain the most widely applied methods [52]. Freeze-drying is based on the removal of water from previously frozen material by sublimation, thereby improving the storage stability of probiotic microorganisms [52,53]. However, dehydration may cause damage to cellular structures and reduce cell viability. To minimize these adverse effects, various cryoprotectants and lyoprotectants, including sugars, prebiotics, and polymeric compounds, are commonly incorporated during the drying process [54]. In contrast, spray-drying involves the conversion of a liquid suspension or solution into a dry powder by atomizing the material into a stream of hot air, enabling rapid moisture removal and efficient production of a stable dry product [55,56].

In addition to drying techniques, encapsulation has emerged as an important strategy for improving the stability of probiotic microorganisms [57]. This technology involves enclosing probiotic cells within a protective coating or matrix, thereby reducing the detrimental effects of environmental factors, storage conditions, and the gastrointestinal environment on microbial viability [58]. The most commonly used encapsulation materials include alginate, chitosan, pectin, gelatin, starch, and various polysaccharides [52,57,58]. Encapsulation enhances the resistance of probiotics to gastric acidity, bile salts, and digestive enzymes, while also improving their stability during storage [57,58,59].

The most widely used encapsulation techniques for probiotics are extrusion and emulsification [60]. In the extrusion method, probiotic microorganisms are mixed with a polymer solution, most commonly sodium alginate, and the resulting suspension is introduced dropwise into a calcium chloride solution, where ionic cross-linking leads to the formation of gel microcapsules [61,62]. Emulsification is based on the formation of microcapsules within a system of immiscible liquids, typically a water-in-oil emulsion, and generally produces smaller microcapsules than extrusion [63].

These technologies enhance probiotic survival during storage and gastrointestinal transit, thereby facilitating more efficient delivery of viable microorganisms to their site of action. Collectively, modern probiotic delivery and protection technologies improve product stability and preserve the functional activity of probiotics throughout storage and administration [63,64].

## 5. Mechanisms of Probiotic Action

**Figure 3:**
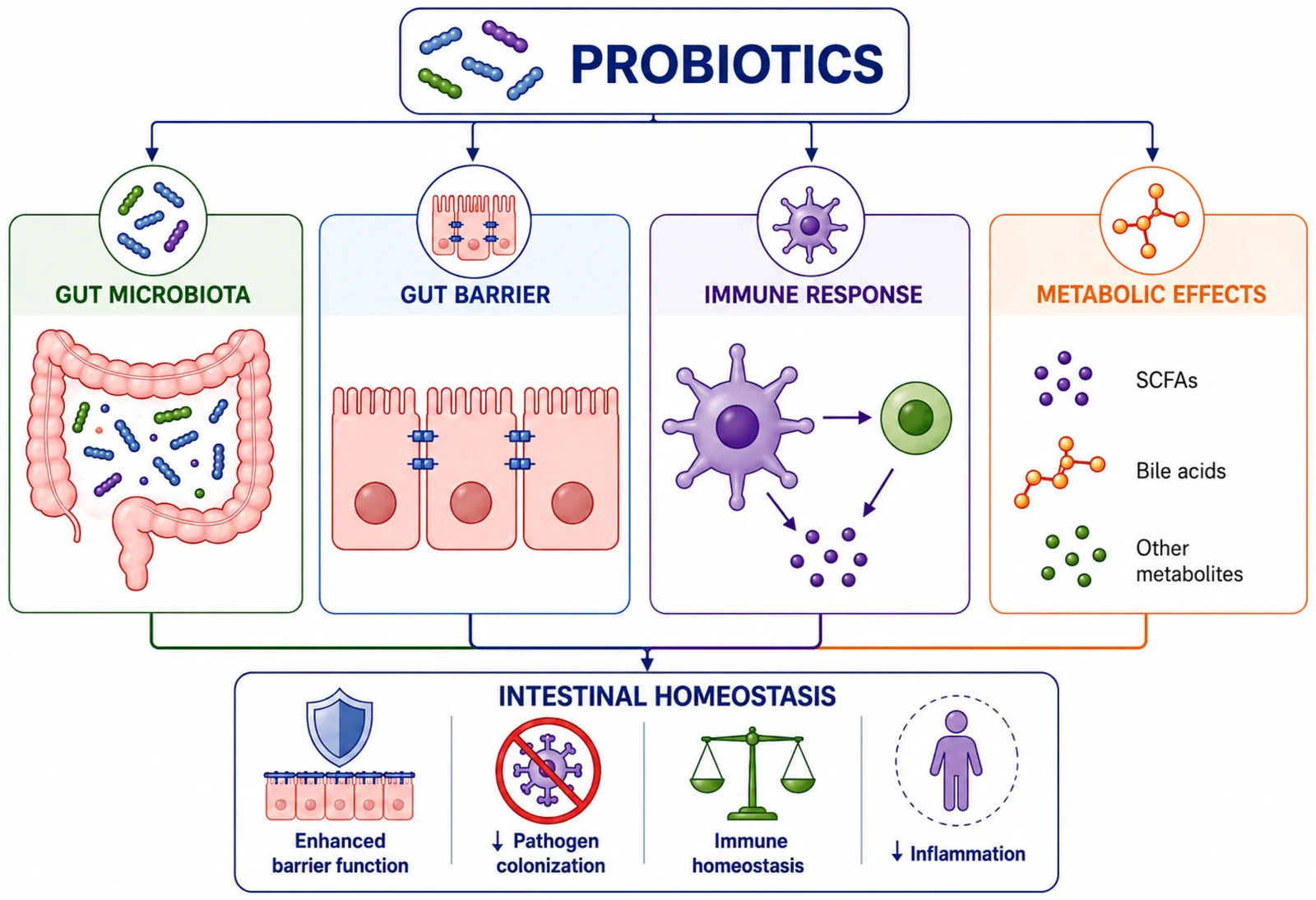
Major mechanisms of probiotic action. Modulation of the gut microbiota, enhancement of intestinal barrier function, immunomodulation, and metabolic effects represent the principal mechanisms through which probiotics contribute to intestinal homeostasis and host health. Arrows indicate the direction of the relationships between probiotic administration, the principal mechanisms of action, and their effects on intestinal homeostasis.

The therapeutic effects of probiotics are mediated through several interconnected mechanisms, including modulation of the gut microbiota, enhancement of intestinal barrier function, immunomodulation, and regulation of host metabolic activity (Figure 3). Probiotics modulate the composition and functional activity of the gut microbiota by promoting the restoration of eubiosis, normalizing disrupted microbial communities, competitively excluding pathogens, and producing organic acids and bacteriocins [65,66,67,68]. In addition, probiotics compete with pathogenic microorganisms for adhesion sites on the intestinal epithelium and available nutrients, thereby preventing pathogen colonization and contributing to the maintenance of microbial homeostasis [65].

Probiotics enhance intestinal barrier integrity by increasing the expression of tight junction proteins, including occludin, claudins, and zonula occludens-1 (ZO-1), stimulating mucin production, and reducing intestinal permeability [66,67]. Selected probiotic strains have been reported in experimental models to modulate NF-κB-, MLCK-, and MAPK-related signaling pathways [69].

Probiotics modulate both innate and adaptive immune responses by regulating cytokine production, inducing regulatory T cells (Tregs), promoting T-cell differentiation, enhancing immunoglobulin A (IgA) secretion, increasing the production of anti-inflammatory cytokines, including interleukin-10 (IL-10) and transforming growth factor-β (TGF-β), and modulating the activity of gut-associated lymphoid tissue (GALT), thereby contributing to intestinal immune homeostasis [66,68].

The metabolic effects of probiotics are largely mediated through the production of short-chain fatty acids (SCFAs) and other bioactive microbial metabolites, which support intestinal barrier integrity, regulate immune responses, and contribute to host metabolic homeostasis [66,67,68]. These mechanisms are closely interconnected and collectively promote intestinal homeostasis, reduce inflammation, and protect against the development of gastrointestinal diseases [66,68,69]. Importantly, the biological effects of probiotics are strain-specific and depend on the particular microorganism used, its dose, duration of administration, and the individual characteristics of the host gut microbiota. These factors should therefore be carefully considered when interpreting the results of clinical studies [66,69].

## 6. Clinical Efficacy of Probiotics: Evidence Across Gastrointestinal and Selected Extraintestinal Disorders

Despite the widespread use of probiotics in clinical practice, their efficacy varies across diseases and should not be regarded as a universal property of all probiotic microorganisms. Current evidence indicates that clinical benefits are largely strain-specific and depend on probiotic formulation, dosage, treatment duration, disease characteristics, and patient-related factors [15,70]. Furthermore, the substantial heterogeneity of published studies hampers direct comparison of results and limits the extrapolation of findings across different probiotic preparations [70,71].

An overview of the current evidence on the clinical efficacy of probiotics in gastrointestinal diseases and emerging extraintestinal applications is presented in Table 1. As shown in the table, the strength of evidence varies considerably across clinical indications. The most robust evidence supports the use of probiotics for the prevention of antibiotic-associated diarrhea and necrotizing enterocolitis, whereas for most other indications the reported clinical benefits remain strain-specific and require further confirmation through high-quality clinical studies.

Antibiotic-associated diarrhea (AAD) is one of the most extensively studied indications for probiotic use and is supported by the strongest body of clinical evidence currently available. Compared with many other clinical indications, the efficacy of probiotics for the prevention of AAD has been consistently demonstrated in multiple trials and meta-analyses, although the magnitude and certainty of benefit vary across strains, populations, and study designs [82]. However, these findings should not be extrapolated to all probiotic preparations. Current evidence indicates that the clinical benefits of probiotics are highly strain-specific, with the most convincing evidence available for *Lacticaseibacillus rhamnosus* GG and *Saccharomyces boulardii*, whereas the efficacy of most other probiotic strains remains insufficiently established [17,83]. The observed variability in clinical outcomes is likely attributable to multiple factors, including strain-specific characteristics, patient-related factors, antibiotic regimens, probiotic dosage and duration, and differences in study design [15,17]. Considerable heterogeneity also exists among randomized controlled trials with respect to the antibiotics administered, patient populations, probiotic formulations, dosages, treatment duration, and outcome definitions. Furthermore, many studies have evaluated multi-strain probiotic formulations, making it difficult to determine the contribution of individual strains to the observed clinical effects [17,74]. Accordingly, current clinical guidelines recommend only those probiotic strains whose efficacy has been demonstrated in well-designed clinical trials and do not consider probiotics as a single therapeutic class [83].

*Clostridioides difficile* infection (CDI) is one of the most serious complications of antibiotic therapy and is associated with substantial morbidity, a high risk of recurrence, prolonged hospitalization, and increased healthcare costs. Antibiotic-induced disruption of the intestinal microbiota is considered the primary risk factor for CDI, as it creates favorable conditions for *C. difficile* colonization and toxin production [84,85]. Consequently, probiotics have been investigated primarily as a preventive strategy aimed at maintaining microbial homeostasis during antibiotic treatment. Despite the large number of published studies, the preventive efficacy of probiotics against CDI remains inconsistent and cannot be considered a universal effect [84,85,86]. Current evidence indicates that clinical efficacy is highly strain-specific. Among the formulations evaluated in clinical studies, *Saccharomyces boulardii* CNCM I-745 and certain multi-strain preparations, including Bio-K+^®^, have received particular attention; however, the overall evidence remains heterogeneous [85,87]. The observed variability in clinical outcomes is likely attributable to both the biological characteristics of individual probiotic strains and the methodological heterogeneity of clinical studies. The proposed mechanisms underlying the preventive effects of these probiotics include restoration of the intestinal microbiota following antibiotic therapy, inhibition of *C. difficile* colonization, reinforcement of intestinal barrier function, and modulation of the host immune response [87]. Moreover, most available studies have focused on a limited number of probiotic strains, restricting the generalizability of these findings to other commercially available probiotic preparations. Accordingly, current clinical guidelines suggest that probiotics for the prevention of CDI may be considered only for specific strains or strain combinations supported by clinical evidence, whereas their use for the treatment of established CDI is not recommended outside the setting of clinical trials [86].

Irritable bowel syndrome (IBS) is one of the most common functional gastrointestinal disorders and is characterized by chronic abdominal pain, altered bowel motility, and changes in bowel habits [88,89]. The current understanding of IBS pathogenesis highlights the important roles of gut microbiota dysbiosis, increased intestinal permeability, low-grade inflammation, visceral hypersensitivity, and dysregulation of the gut–brain axis, providing the rationale for the use of probiotics as an adjunctive therapeutic approach [88,90]. Evidence from numerous randomized controlled trials and meta-analyses suggests that probiotics may alleviate the major symptoms of IBS, including abdominal pain, bloating, and overall symptom severity [73]. However, clinical efficacy varies substantially across studies, with the most convincing evidence reported only for selected strains, including *Lactiplantibacillus plantarum* 299v, *Bifidobacterium infantis* 35624, *Bifidobacterium bifidum* MIMBb75, and certain multi-strain probiotic formulations [73,90]. These findings further support the concept that the therapeutic effects of probiotics are predominantly strain-specific and should not be generalized to all probiotic microorganisms [73,89]. One of the major reasons for the inconsistent findings is the marked clinical heterogeneity of IBS itself. The disorder comprises several clinical subtypes, including diarrhea-predominant, constipation-predominant, mixed, and unclassified IBS, which differ in their underlying pathophysiological mechanisms and gut microbiota composition [89,90]. These differences may partly explain why the same probiotic strain demonstrates variable efficacy across different patient populations. Furthermore, considerable heterogeneity exists among studies with respect to probiotic formulations, dosages, treatment duration, outcome measures, and patient characteristics, making direct comparison of findings challenging. It should also be noted that most studies have primarily evaluated improvements in clinical symptoms, whereas the effects of probiotics on the key pathophysiological mechanisms underlying IBS remain insufficiently understood [88,89]. Overall, despite encouraging findings from individual randomized controlled trials and meta-analyses, the use of probiotics for IBS remains controversial. The selection of probiotic therapy should be based on clinical evidence supporting the efficacy of specific strains. Accordingly, the American College of Gastroenterology (ACG) currently recommends against the routine use of probiotics for the treatment of global IBS symptoms because of the very low certainty of the available evidence and the substantial heterogeneity among published studies [75,89,90].

Inflammatory bowel disease (IBD), encompassing ulcerative colitis (UC) and Crohn’s disease (CD), comprises a group of chronic immune-mediated inflammatory disorders resulting from complex interactions among genetic susceptibility, immune dysregulation, gut microbiota alterations, and environmental factors. Because dysbiosis is considered a key component of IBD pathogenesis, probiotics have attracted considerable interest as an adjunctive therapeutic approach aimed at restoring microbial homeostasis and modulating intestinal inflammation [91,92,93]. Despite shared pathogenic mechanisms, the clinical efficacy of probiotics differs substantially between UC and CD [86,93]. Current evidence indicates that the strongest support for probiotic use is available for UC [91,93]. Several randomized controlled trials and meta-analyses have demonstrated that selected probiotic preparations, particularly the De Simone formulation and *Escherichia coli* Nissle 1917, may provide clinical benefits in UC, especially when used as adjuncts to standard therapy [91,92,93]. In contrast, most studies in CD have failed to demonstrate significant benefits of probiotics for either the induction or maintenance of remission, suggesting a considerably more limited therapeutic role in this condition. The observed differences in efficacy are likely related to disease-specific pathogenic mechanisms [92,93]. In UC, inflammation is primarily confined to the colonic mucosa, allowing probiotic microorganisms to interact directly with the intestinal epithelium and resident microbiota [86,92,93]. By contrast, CD is characterized by transmural inflammation and frequently involves the small intestine, which may limit the ability of probiotics to influence key pathogenic processes [91,93]. Nevertheless, the lack of convincing clinical efficacy in CD may also reflect the limited statistical power of many studies and the substantial clinical heterogeneity of the enrolled patient populations [92,93]. Interpretation of the available evidence is further complicated by considerable clinical and methodological heterogeneity across published studies [91,92,93]. Important differences exist in probiotic strains and formulations, dosages, treatment duration, disease activity, concomitant therapies, and the clinical outcomes assessed [91,93]. Furthermore, many studies included relatively small patient cohorts, limiting their ability to detect modest treatment effects and preventing definitive conclusions regarding which probiotic strains or patient subgroups are most likely to benefit [92,93]. Taken together, although selected probiotic preparations appear promising as adjunctive therapy for UC, the available evidence remains insufficiently consistent to support their widespread clinical use. In accordance with current American Gastroenterological Association (AGA) guidelines, probiotics for both UC and CD should therefore be considered only within the context of clinical trials until more robust evidence of efficacy becomes available [86].

Acute infectious diarrhea remains one of the leading causes of gastroenteritis worldwide, particularly among young children, and continues to contribute substantially to global morbidity and hospitalization rates. Although oral rehydration therapy and correction of fluid and electrolyte imbalance remain the cornerstone of treatment, increasing attention has been directed toward the use of probiotics as an adjunctive therapeutic strategy [94]. The potential clinical benefits of selected probiotic strains are thought to result from their ability to modulate the gut microbiota, reinforce intestinal barrier function, and regulate host immune responses [95]. Evidence from recent systematic reviews and meta-analyses suggests that selected probiotic strains may reduce the duration of diarrhea and accelerate clinical recovery. However, the magnitude of these benefits is generally modest and varies considerably across studies [94,96]. The most extensively investigated probiotics include *Saccharomyces boulardii* CNCM I-745, *Lacticaseibacillus rhamnosus* GG, *Limosilactobacillus reuteri* DSM 17938, and *Bacillus clausii*, whereas convincing evidence for most other probiotic microorganisms remains limited [94]. Interpretation of the available evidence is further complicated by substantial heterogeneity in patient age, diarrhea etiology (viral or bacterial), disease severity, probiotic strains, dosages, treatment duration, and outcome measures [94,96]. Moreover, several large randomized controlled trials have failed to demonstrate significant benefits of probiotics compared with placebo, contributing to the more cautious recommendations issued by contemporary international clinical guidelines regarding their routine use. According to the European Society for Paediatric Gastroenterology, Hepatology and Nutrition (ESPGHAN), probiotics for acute gastroenteritis may be considered only for selected clinically evaluated strains, including *Saccharomyces boulardii* CNCM I-745, *Lacticaseibacillus rhamnosus* GG, and *Limosilactobacillus reuteri* DSM 17938, whereas the routine use of probiotic preparations without demonstrated strain-specific efficacy is not recommended [94].

Compared with adults, the gut microbiota of children is considerably more dynamic, making it both more susceptible to environmental influences and more amenable to modulation by probiotic microorganisms [97]. At present, the strongest evidence supports the use of probiotics for the management of infantile colic, the prevention of necrotizing enterocolitis (NEC) in preterm infants, and selected cases of acute infectious diarrhea. However, the clinical effects of probiotics are predominantly strain-specific and depend on the underlying condition, the age of the child, and the quality of the available evidence; therefore, findings should not be generalized to other probiotic preparations [94].

For infantile colic, the most consistent benefits have been demonstrated in breastfed infants, whereas convincing evidence of efficacy in formula-fed infants remains limited [76,98]. Moreover, the majority of clinical studies have evaluated *Limosilactobacillus reuteri* DSM 17938, and these findings should not be extrapolated to other probiotic strains [94].

In children with acute infectious diarrhea, selected probiotic strains may reduce the duration of illness and accelerate clinical recovery; however, the magnitude of the benefit is generally modest and varies considerably across studies. The most extensively investigated strains include *Saccharomyces boulardii* CNCM I-745, *Lacticaseibacillus rhamnosus* GG, and *Limosilactobacillus reuteri* DSM 17938, although the overall evidence remains heterogeneous [94,98,99]. Interpretation of the available evidence is further complicated by differences in patient age, infection etiology, disease severity, probiotic formulations, and outcome measures [96,100].

The strongest evidence in pediatric practice is available for the prevention of NEC in preterm infants. Recent systematic reviews and network meta-analyses have demonstrated that selected probiotic preparations significantly reduce the incidence of NEC and all-cause mortality. The greatest benefits have been observed with multi-strain formulations containing combinations of *Bifidobacterium* and *Lactobacillus* species and, in some studies, *Enterococcus* species. Current evidence suggests that selected probiotic preparations may reduce the risk of NEC and mortality in preterm infants, although the magnitude of benefit and the optimal strain or strain combination remain uncertain [101,102].

Beyond gastrointestinal disorders, interest in the potential therapeutic applications of probiotics in extraintestinal diseases has increased substantially in recent years. This interest has been driven by growing evidence highlighting the central role of the gut microbiota in regulating immune, metabolic, and neuroendocrine processes [103,104,105]. It has been proposed that modulation of the gut microbiota may exert systemic effects extending beyond the gastrointestinal tract. However, despite the large number of published studies, the evidence supporting most extraintestinal indications remains limited and is generally less robust than that for established gastrointestinal applications. This is largely attributable to considerable clinical and methodological heterogeneity, including differences in probiotic strains, treatment regimens, study populations, and clinical endpoints [70,71,106].

Among extraintestinal indications, atopic dermatitis is the most extensively investigated. Probiotics are thought to alleviate allergic inflammation by modulating the gut microbiota, enhancing intestinal barrier function, and regulating immune responses, including restoration of the balance between T-helper cell subsets and promotion of regulatory T-cell (Treg) activity [107,108]. Meta-analyses suggest that probiotics may reduce the severity of atopic dermatitis, with the strongest evidence reported in pediatric populations [109,110]. Nevertheless, clinical efficacy appears to depend substantially on the probiotic strain used, treatment duration, and patient characteristics [107,110].

In addition to allergic diseases, the potential role of probiotics in the management of metabolic disorders, including obesity and metabolic syndrome, has attracted increasing attention. This interest is supported by accumulating evidence linking the gut microbiota to energy homeostasis, chronic low-grade inflammation, and insulin resistance [103,105]. Although certain probiotic strains have demonstrated modest improvements in anthropometric and metabolic parameters, such as reductions in waist circumference and low-density lipoprotein cholesterol levels, the magnitude of these effects depends on treatment duration and population characteristics and remains of limited clinical significance. Taken together, the available evidence suggests that probiotics may currently be considered as an adjunct to comprehensive lifestyle and pharmacological interventions rather than as a standalone therapeutic strategy [111].

Another rapidly evolving area of research concerns the influence of probiotics on central nervous system function through the gut–brain axis. Particular attention has been focused on psychobiotics, a subgroup of probiotic microorganisms capable of influencing brain function via modulation of the gut–brain axis [112]. Their proposed mechanisms include alterations in gut microbial composition, attenuation of systemic inflammation, modulation of short-chain fatty acid and neuroactive metabolite production, and regulation of the hypothalamic–pituitary–adrenal axis [113]. Recent meta-analyses indicate that selected probiotic strains may reduce symptoms of anxiety and depression; however, the observed effects are strain-specific and remain heterogeneous across studies. Consequently, the current evidence is insufficient to support psychobiotics as a replacement for standard evidence-based treatments for mental disorders [114].

Taken together, current evidence indicates that the clinical efficacy of probiotics is not a universal property shared by all probiotic microorganisms but is largely determined by the specific strain, the disease being treated, patient characteristics, dosage, and treatment duration [15,20,70]. Despite the substantial body of published research, direct comparison of study findings remains challenging because of considerable heterogeneity in study design, probiotic formulations, and clinical outcome measures. Nevertheless, the available evidence suggests that probiotics with demonstrated strain-specific efficacy may be effective for the prevention of selected diseases or as adjunctive therapy in specific clinical settings [20,70]. However, probiotics should not currently be regarded as a substitute for established standard-of-care treatments. Their clinical use should be guided by current evidence-based recommendations and supported by high-quality clinical studies demonstrating the efficacy and safety of specific probiotic strains for well-defined clinical indications [15,86,94].

## 7. Safety of Probiotic Preparations: Adverse Effects, Risk Factors, and Quality Considerations

Despite the widespread clinical use of probiotics and their long history of application, their safety profile continues to be the subject of active investigation. Current clinical trials and systematic reviews consistently indicate that probiotic administration is generally not associated with an increased risk of serious adverse events compared with control groups, supporting their overall favorable safety profile [5,23]. Nevertheless, these findings should be interpreted with caution, as safety was not the primary endpoint in many clinical studies, and considerable variability exists in the methods used to identify, classify, and report adverse events, limiting the accuracy of risk estimation [23]. The most commonly reported adverse events are mild gastrointestinal symptoms, including flatulence, abdominal bloating, and mild abdominal discomfort [5]. Randomized controlled trials have shown that the incidence of these symptoms is generally comparable to that observed in placebo groups. Moreover, they typically occur during the initial stages of treatment, are transient in nature, and rarely require discontinuation of probiotic therapy [26,69].

The likelihood of developing such symptoms may vary according to the probiotic strain, administered dose, and individual patient characteristics. However, their clinical interpretation remains challenging because adverse events have not been assessed or reported in a standardized manner across studies [115]. Taken together, the available evidence does not indicate a clinically meaningful deterioration in the overall safety profile of probiotics as a result of these mild gastrointestinal adverse events [70].

Probiotic-associated infections have been reported most frequently following the administration of microorganisms belonging to the genera *Lactobacillus* and *Saccharomyces*. Among bacterial probiotics, most published cases involve *Lacticaseibacillus rhamnosus* GG, *Lacticaseibacillus paracasei*, *Lactiplantibacillus plantarum*, and other lactic acid bacteria, whereas the majority of fungal probiotic-associated infections have been attributed to *Saccharomyces boulardii* [29,116]. The true incidence of probiotic-associated infections remains difficult to determine because of the lack of standardized diagnostic criteria, differences in pharmacovigilance systems, and the fact that the available evidence is derived predominantly from isolated case reports and small case series rather than systematic surveillance studies [23,29,116,117].

**Figure 4:**
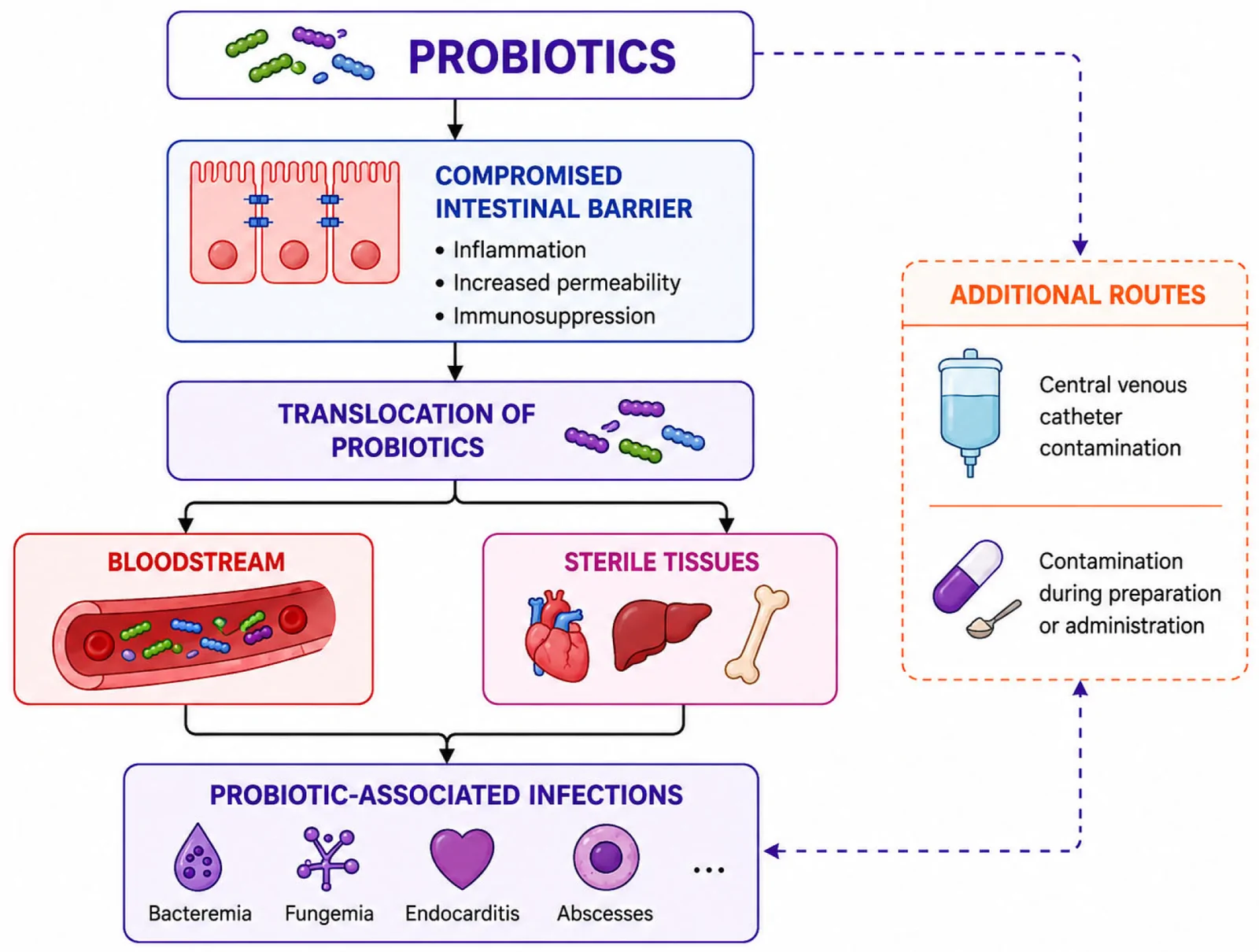
Proposed mechanisms and potential routes of probiotic-associated infections. Schematic overview of the principal mechanisms underlying probiotic-associated infections, including microbial translocation across a compromised intestinal barrier and contamination during probiotic preparation, administration, or central venous catheter handling.

Despite the generally favorable safety profile of probiotics, rare cases of infectious complications associated with probiotic microorganisms have been described. The most clinically significant manifestations include bacteremia, fungemia, infective endocarditis, localized abscesses, and other invasive infections resulting from the dissemination of probiotic microorganisms beyond the gastrointestinal tract. Although these events are exceedingly uncommon, they may have serious clinical consequences in patients with predisposing risk factors and therefore warrant careful consideration when probiotic therapy is prescribed. The principal mechanism underlying probiotic-associated infections is believed to be microbial translocation across the intestinal barrier into the bloodstream or other normally sterile tissues (Figure 4) [23,118,119,120]. Under physiological conditions, the intestinal epithelium, mucus layer, and mucosal immune defenses provide an effective barrier that limits microbial translocation [121,122]. However, impairment of intestinal barrier integrity, severe mucosal inflammation, increased intestinal permeability, or immunosuppression may facilitate the translocation of probiotic microorganisms. Additional mechanisms include contamination of central venous catheters as well as contamination during the preparation or administration of probiotic products [23,118].

Among the reported infectious complications, bacteremia associated with probiotic strains such as *Lacticaseibacillus rhamnosus* GG, *Lacticaseibacillus paracasei*, *Lactiplantibacillus plantarum*, and other members of the former *Lactobacillus* genus has been described most frequently. In the majority of published cases, bacteremia occurred in patients with severe underlying diseases, profound immunosuppression, short bowel syndrome, inflammatory bowel disease, recent gastrointestinal surgery, or prolonged use of central venous catheters. However, establishing a definitive causal relationship between probiotic administration and subsequent infection is often challenging, as genetically similar microorganisms may also be present as part of the normal intestinal microbiota or acquired independently of probiotic use. Consequently, molecular strain typing is required to confirm the origin of the infecting organism [29].

Another clinically important complication is fungemia associated with the yeast probiotic *Saccharomyces boulardii*. In contrast to bacterial probiotics, cases of *S. boulardii* fungemia have been relatively well documented and have been reported predominantly in critically ill patients, preterm neonates, individuals with severe underlying diseases, and patients with central venous catheters [116]. Infection is thought to occur either through microbial translocation across a compromised intestinal barrier or through exogenous contamination of central venous catheters during the opening, preparation, or administration of probiotic products [116,123].

**Figure 5:**
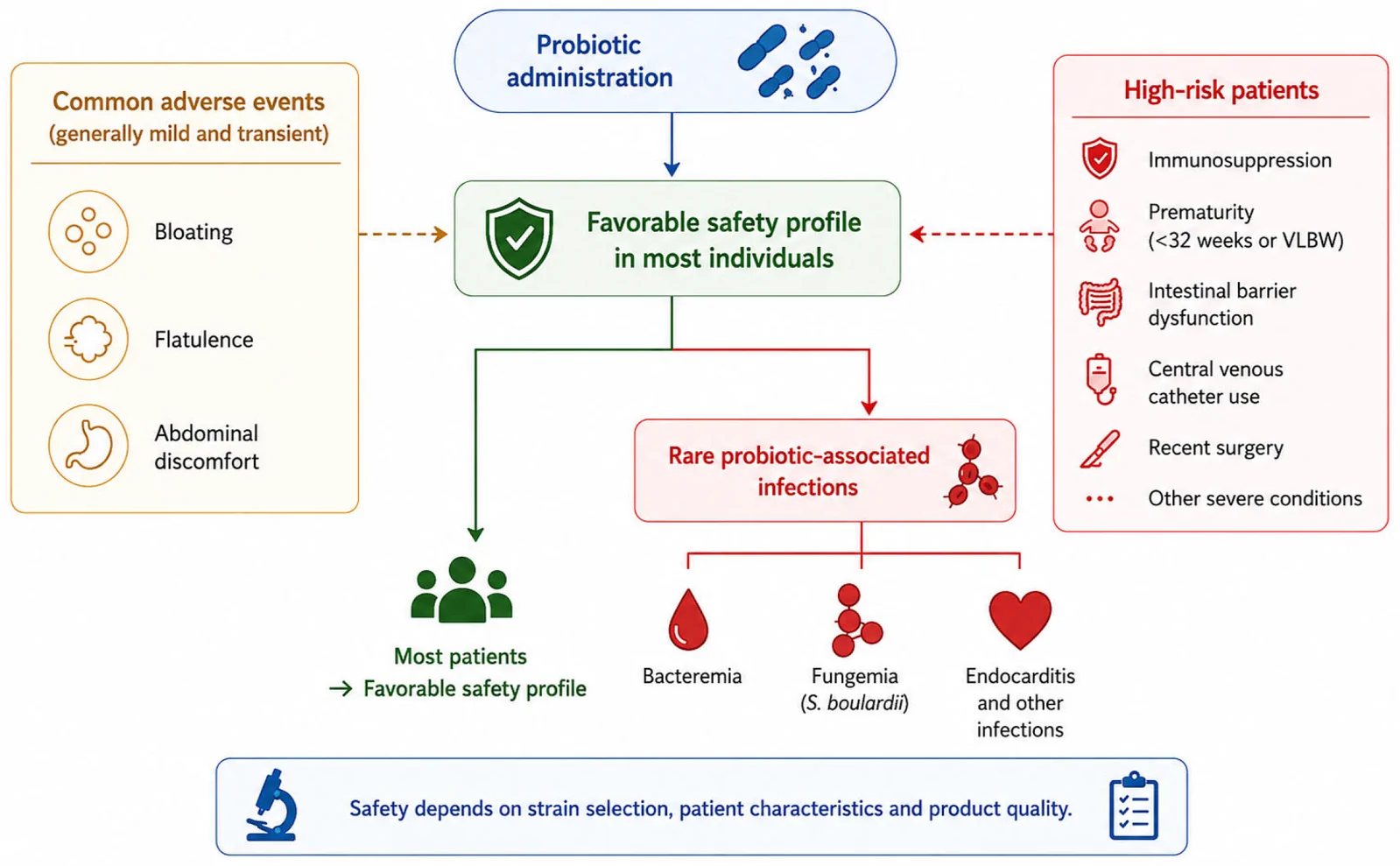
Overview of the safety profile of probiotic therapy. Schematic overview of the overall safety profile of probiotics, including commonly reported mild gastrointestinal adverse events, patient populations at increased risk of probiotic-associated infections, and the rare infectious complications reported in clinical practice. Arrows indicate the direction of the relationships between probiotic administration, patient-related risk factors, safety outcomes, and potential adverse events or infectious complications.

In addition to bacteremia and fungemia, isolated cases of infective endocarditis, peritonitis, urinary tract infections, liver abscesses, and other localized infections caused by probiotic microorganisms have also been reported [29,117]. Overall, the available evidence indicates that probiotic-associated infections represent exceptionally rare complications of probiotic therapy [23,29,117]. Nevertheless, although probiotics exhibit a favorable safety profile in the general population, certain patient groups appear to be at substantially higher risk of developing infectious complications (Figure 5). Most reported cases of probiotic-associated bacteremia, fungemia, and other invasive infections have occurred in individuals with severe impairment of host immune defenses, disruption of intestinal barrier integrity, or serious underlying diseases, suggesting that host-related factors play a major role, although strain characteristics, product quality, dose, and route of administration may also contribute [29,117].

Patients with immunocompromising conditions represent one of the principal high-risk groups for probiotic-associated infections. This population includes individuals with malignancies receiving chemotherapy, recipients of solid organ or hematopoietic stem cell transplantation, patients undergoing prolonged treatment with systemic corticosteroids or other immunosuppressive agents, and those with congenital or acquired immunodeficiency disorders [124,125]. In immunocompromised individuals, impaired microbial clearance together with compromised mucosal barrier function may increase the risk of microbial translocation and subsequent systemic infection [126]. Although such complications remain rare, probiotic use in these patients requires careful individual assessment of the potential benefits and risks [125,126].

Patients with severe disruption of intestinal barrier integrity also represent a high-risk population [127,128]. Intestinal barrier dysfunction may occur in the setting of active inflammatory bowel disease, short bowel syndrome, intestinal ischemia, or following major gastrointestinal surgery [127]. Under these conditions, increased intestinal permeability may facilitate microbial translocation across the intestinal mucosa [129].

An additional mechanism of probiotic-associated infection has been described in patients with central venous catheters and is independent of microbial translocation across the intestinal barrier. Contamination of the surrounding environment or the hands of healthcare personnel during the preparation and administration of probiotic products may result in subsequent contamination of central venous catheters [29]. This mechanism has been most extensively documented for *Saccharomyces boulardii*. Accordingly, probiotic capsules should not be opened and probiotic suspensions should not be prepared in close proximity to patients with central venous catheters, and strict aseptic precautions should be observed during probiotic preparation and administration [123].

Preterm neonates, particularly those with extremely low birth weight, constitute another vulnerable population. Immaturity of the immune system, increased intestinal permeability, the need for invasive medical procedures, and prolonged parenteral or enteral nutritional support may predispose these infants to systemic infections [130]. Although numerous clinical studies have demonstrated that specific probiotic strains reduce the incidence of necrotizing enterocolitis and mortality in preterm infants, isolated cases of probiotic-associated bacteremia and fungemia have also been reported in this population [130,131,132]. Therefore, probiotic administration in preterm neonates should be restricted to products with well-established safety and clinical efficacy and implemented only under rigorous quality assurance and infection control measures [133].

Particular caution is also warranted when considering probiotic therapy in patients admitted to intensive care units (ICUs). These patients frequently present with multiple predisposing factors for infection, including severe underlying illness, impaired immune function, prolonged exposure to broad-spectrum antibiotics, the presence of vascular catheters, mechanical ventilation, and disruption of intestinal barrier integrity [29,117]. Although probiotic-associated infectious complications remain uncommon, findings from individual clinical studies and case reports suggest that probiotics should be administered with caution in critically ill patients [23,29].

The potential transfer of antibiotic resistance genes by probiotic microorganisms has attracted increasing attention in recent years. Although the majority of commercially available probiotic strains are considered safe and are routinely evaluated for virulence factors and antimicrobial resistance, the possibility of horizontal gene transfer between probiotic and pathogenic microorganisms cannot be completely excluded [26,134]. Particular concern has focused on mobile genetic elements, including plasmids, transposons, and integrons, which are recognized as the principal vehicles for the dissemination of antibiotic resistance genes among bacterial species. Horizontal gene transfer may occur through conjugation, transformation, or transduction and represents one of the major mechanisms underlying the spread of antimicrobial resistance [135]. Theoretically, probiotic microorganisms carrying transferable resistance determinants may act as reservoirs of these genes within the intestinal microbiota, thereby facilitating their transmission to opportunistic or pathogenic bacteria [119,134]. Genes conferring resistance to tetracyclines, macrolides, aminoglycosides, and glycopeptides have received particular attention because they are among the most frequently detected resistance determinants in lactic acid bacteria [134,135].

Importantly, not all forms of antibiotic resistance have the same clinical significance. Intrinsic resistance in probiotic strains is generally considered non-transferable and therefore does not represent the same safety concern as acquired resistance associated with mobile genetic elements. The latter is of greater clinical relevance because of its potential for horizontal transfer within the intestinal microbiota [134]. Consequently, probiotic strains intended for clinical or commercial use should be screened for transferable antimicrobial resistance determinants and other relevant safety characteristics [26].

The safety of probiotic preparations depends not only on the biological characteristics of the microorganisms they contain but also on compliance with manufacturing standards, product quality, and effective regulatory oversight [26,120,136]. In many countries, a substantial proportion of probiotic products are marketed as dietary supplements rather than medicinal products, and regulatory requirements governing their manufacture, quality control, and labeling vary considerably across jurisdictions and product categories [136]. Consequently, commercially available probiotic products may differ substantially in microbial composition, strain identity, viability, and overall quality, potentially affecting both their safety and clinical efficacy [21,136,137]. Common quality-related concerns include discrepancies between the labeled and actual microbial composition, lower viable cell counts than declared by manufacturers, strain misidentification, microbial contamination, and inadequate storage conditions leading to reduced viability of probiotic cultures [136,137]. Because the safety and clinical efficacy of probiotics are strain-specific, accurate strain identification using modern molecular methods, together with screening for potentially harmful genetic determinants, is essential [26,120]. Collectively, these considerations highlight the importance of rigorous quality control throughout the development, manufacturing, storage, and distribution of probiotic products [120,136,137].

In recent decades, increasing attention has been devoted to the quality and safety of probiotic products by international scientific organizations and regulatory authorities [138]. Current evaluation criteria include accurate strain identification, confirmation of the absence of transferable antibiotic resistance genes and virulence factors, maintenance of microbial viability throughout the product shelf life, and demonstration of both safety and clinical efficacy in well-designed clinical studies conducted according to internationally accepted scientific standards [26]. Nevertheless, regulatory requirements for probiotic products continue to vary across countries and product categories, highlighting the challenges associated with establishing consistent quality and safety standards [138].

## 8. Future Perspectives

Despite substantial progress in understanding the mechanisms of probiotic action and the accumulation of extensive experimental and clinical evidence, several fundamental questions remain unresolved. Although numerous randomized controlled trials and meta-analyses have demonstrated the efficacy of specific probiotic strains for selected diseases, the available evidence remains inconsistent for many gastrointestinal disorders and particularly extraintestinal conditions. This variability is likely attributable to considerable clinical and methodological heterogeneity among studies, including differences in probiotic formulations, patient populations, treatment regimens, and outcome measures, as well as the complexity of interactions between the gut microbiota and the host [20,95,120].

One of the major priorities for future research is to identify the factors that determine individual responses to probiotic therapy. Emerging evidence suggests that clinical efficacy may be influenced by the baseline composition of the gut microbiota, intestinal barrier integrity, host immune responses, and other patient-specific characteristics [120,139]. Accordingly, the development of patient stratification strategies based on microbiome, immunological, metabolomic, and other molecular biomarkers represents an important research direction. Future studies should also seek to identify patient subgroups that are most likely to benefit from specific probiotic strains, thereby facilitating the transition from empirical probiotic supplementation toward more personalized microbiome-based interventions [140].

Further progress in this field will require large, multicenter randomized controlled trials using genetically well-characterized probiotic strains, standardized manufacturing processes, harmonized clinical outcome measures, and long-term patient follow-up. Greater emphasis should also be placed on improving methodological consistency through transparent reporting of probiotic strain identity, viable cell counts, treatment regimens, and clinically relevant outcome measures to improve comparability and reproducibility across studies. Equally important will be the implementation of standardized quality assessment procedures for probiotic products, confirmation of genetic stability, and comprehensive long-term safety evaluations, particularly in high-risk patient populations [120,141]. The principal challenges and research priorities for advancing probiotic therapy are summarized in Table 2.

In the longer term, integrating gut microbiota data with clinical, immunological, and metabolomic characteristics may facilitate the development of more rational and personalized approaches to probiotic therapy [139]. Beyond improving scientific evidence, coordinated international efforts to improve consistency in quality standards, regulatory approaches, and strain characterization will be essential for ensuring the safety, consistency, and reproducibility of probiotic products used in both clinical practice and research [120,141]. Collectively, these advances have the potential to improve treatment efficacy, enhance the reproducibility of clinical research findings, and support the broader implementation of evidence-based probiotic therapy within the framework of personalized medicine.

## 9. Conclusions

This review demonstrates that probiotics possess considerable therapeutic potential; however, they should not be considered a single therapeutic entity. Their clinical effects are largely strain-specific and are further influenced by the disease being treated, patient characteristics, dosage, treatment duration, and formulation. Consequently, evidence obtained for one probiotic strain cannot be extrapolated to other strains or commercial formulations, even when they belong to the same microbial species.

The available clinical literature also highlights substantial differences in the maturity of evidence across therapeutic areas. While some applications are supported by relatively consistent clinical data, many gastrointestinal and particularly extraintestinal indications are characterized by substantial clinical and methodological heterogeneity. Importantly, uncertainty regarding particular applications should not necessarily be interpreted as evidence of absence of therapeutic potential. Rather, it frequently reflects differences in probiotic strains and combinations, formulations, dosages, treatment duration, study populations, comparator groups, and clinical outcome measures. These sources of heterogeneity substantially limit direct comparison between studies and the generalizability of their findings.

Safety assessment likewise requires consideration of both the probiotic preparation and the clinical context in which it is administered. Although serious probiotic-associated infections are uncommon, their occurrence emphasizes the importance of careful patient selection, particularly in individuals with immunodeficiency, impaired intestinal barrier integrity, central venous catheters, or severe underlying conditions. In addition to patient-related risks, important unresolved concerns include variability in the quality of commercially available products, the potential dissemination of transferable antimicrobial resistance genes, and the absence of internationally harmonized standards for probiotic quality control and safety assessment.

From a clinical perspective, probiotic therapy should therefore be based on evidence obtained for the specific strain or formulation under consideration rather than on generalized assumptions regarding probiotics as a therapeutic class. Preference should be given to preparations and indications supported by well-designed clinical trials and contemporary evidence-based recommendations, while particular caution is warranted in vulnerable populations. Future research should focus on adequately powered, strain-specific clinical trials using standardized outcome measures and clearly characterized probiotic preparations, alongside continued efforts to harmonize regulatory requirements, manufacturing standards, and quality-control procedures.

## Figures and Tables

**Table 1:** Clinical efficacy of probiotic preparations across gastrointestinal diseases and emerging clinical applications.

Disease	Study Design	Probiotic Strain(s)	Duration	Main Findings	Clinical Implications	Limitations	References
**Gastrointestinal disorders**							
Antibiotic-associated diarrhea	Meta-analysis of 17 RCTs (3631 participants)	*Lacticaseibacillus rhamnosus* GG; *Saccharomyces boulardii*; *Lactobacillus acidophilus* La-5 + *Bifidobacterium animalis* subsp. *lactis* Bb-12; representative *Lactobacillus*- and *Bifidobacterium*-based formulations	During antibiotic therapy	Probiotic supplementation reduced the incidence of AAD by 51% (RR 0.49; 95% CI 0.36–0.66), with the strongest evidence for *Lacticaseibacillus rhamnosus* GG and *Saccharomyces boulardii*. No significant increase in adverse events was observed.	Probiotics may be recommended as adjunctive therapy for the prevention of AAD, particularly in patients receiving antibiotics who are at increased risk of diarrhea.	Moderate heterogeneity, variable probiotic regimens, and limited strain-specific evidence.	[72]
*Clostridioides difficile* infection	Strain-specific meta-analysis of RCTs	*Saccharomyces boulardii* CNCM I-745; Bio-K+^®^ (*Lactobacillus acidophilus* CL1285, *Lacticaseibacillus casei* LBC80R, *Lacticaseibacillus rhamnosus* CLR2)	During antibiotic therapy	*Saccharomyces boulardii* CNCM I-745 and the Bio-K+^®^ probiotic mixture demonstrated preventive efficacy against primary CDI, whereas evidence for other probiotic strains remained limited or inconsistent.	Selected probiotic strains may be considered for primary CDI prevention in patients receiving antibiotics; however, efficacy is strain-specific.	Limited number of strain-specific RCTs and insufficient evidence for many probiotic strains.	[20]
Irritable bowel syndrome	Meta-analysis of 82 RCTs (10,332 participants)	*Lactiplantibacillus plantarum* 299V; *Bifidobacterium infantis* 35624; *Bifidobacterium bifidum* MIMBb75; *Saccharomyces boulardii*; representative *Lactobacillus*- and *Bifidobacterium*-based multi-strain formulations	4–12 weeks	Probiotics improved global IBS symptoms, abdominal pain, and bloating/distension; however, the effects were strain- and symptom-specific, with low to very low certainty of evidence.	Selected probiotic strains may be considered as adjunctive therapy for symptom relief, particularly for global symptoms and abdominal pain.	High heterogeneity, predominance of low-quality studies, publication bias, and strain-specific variability.	[73]
Ulcerative colitis	Overview of systematic reviews and meta-analysis (45 RCTs)	De Simone formulation (8-strain probiotic mixture); *Escherichia coli* Nissle 1917; representative *Lactobacillus*- and *Bifidobacterium*-based formulations	4–104 weeks	Probiotics significantly increased clinical remission (OR 2.00; 95% CI 1.28–3.11) and endoscopic remission (OR 2.38; 95% CI 1.41–4.01). A trend toward reduced clinical recurrence was also observed. Multi-strain formulations, particularly the De Simone formulation, demonstrated the greatest efficacy.	Probiotics may be considered as adjunctive therapy for induction and maintenance of remission in mild-to-moderate UC, particularly in combination with 5-ASA.	Considerable heterogeneity in probiotic strains, treatment regimens, and study design; overall certainty of evidence was low.	[74]
Crohn’s disease	Overview of systematic reviews and meta-analysis (45 RCTs)	De Simone formulation (8-strain probiotic mixture); *Saccharomyces boulardii*; *Escherichia coli* Nissle 1917; representative *Lactobacillus*-based formulations	4–52 weeks	Probiotics did not significantly improve induction or maintenance of clinical remission. Current evidence does not support their routine use in patients with Crohn’s disease.	Current evidence does not support the routine use of probiotics for induction or maintenance of remission in Crohn’s disease.	Considerable heterogeneity in probiotic formulations and study quality; overall certainty of evidence was low to very low.	[74]
Acute infectious diarrhea	Meta-analysis of 98 RCTs (17,236 participants)	*Saccharomyces boulardii*; *Lacticaseibacillus rhamnosus*; *Limosilactobacillus reuteri*; *Bacillus clausii*; representative *Lactobacillus*- and *Bifidobacterium*-based formulations	5–14 days	Probiotics increased the likelihood of clinical cure (RR 1.21; 95% CI 1.12–1.30) and reduced diarrhoea duration by 13.3 h. However, the certainty of evidence was low and substantial heterogeneity was observed.	Selected probiotic strains may provide modest symptomatic benefit in children with acute infectious diarrhea; however, current evidence is insufficient to support routine use.	High heterogeneity in probiotic strains and treatment regimens, with low certainty of evidence.	[75]
Infantile colic	Individual participant data meta-analysis of 4 RCTs (345 infants)	*Limosilactobacillus reuteri* DSM 17938	21 days	*Limosilactobacillus reuteri* DSM 17938 significantly reduced crying/fussing time (−25.4 min/day) and increased treatment success. The beneficial effects were demonstrated primarily in breastfed infants.	*Limosilactobacillus reuteri* DSM 17938 may be recommended for the management of infantile colic in breastfed infants.	Only four RCTs were included, and evidence remains insufficient for formula-fed infants.	[76]
Necrotizing enterocolitis (NEC)	Bayesian network meta-analysis of 51 RCTs (11,661 preterm infants)	Representative *Lactobacillus*, *Bifidobacterium*, and *Saccharomyces* spp.; selected multi-genus combinations (*Bifidobacterium* + *Lactobacillus* ± *Enterococcus/Streptococcus*)	From initiation of enteral feeding until hospital discharge	Most probiotic regimens reduced the incidence of NEC at or beyond Bell stage II. The *Bifidobacterium* + *Lactobacillus* + *Enterococcus* combination ranked highest for NEC prevention. Selected probiotic regimens also significantly reduced all-cause mortality, whereas no significant effect on culture-confirmed sepsis was observed.	Selected probiotic formulations may reduce NEC and mortality in preterm infants. Clinical use should be based on well-characterized formulations with established neonatal safety.	Considerable heterogeneity in probiotic formulations, treatment protocols, and patient characteristics; most analyses were performed at the genus rather than strain level.	[77]
**Emerging clinical applications**							
Atopic dermatitis	Meta-analysis of 25 RCTs (14 included in quantitative synthesis)	*Lacticaseibacillus rhamnosus* GG; *Lacticaseibacillus paracasei*; *Lactiplantibacillus plantarum*; *Limosilactobacillus reuteri*; *Bifidobacterium breve* M-16V; *Bifidobacterium longum* BB536; representative multi-strain formulations	4–24 weeks	Probiotic supplementation was associated with a moderate-to-large reduction in SCORAD scores (Hedges’ g = −0.65), whereas no significant improvement in serum IgE levels was observed.	Selected probiotic strains may be considered as adjunctive therapy for reducing disease severity in pediatric atopic dermatitis; however, the effects remain strain-specific.	High heterogeneity (I^2^ = 92%) and substantial variation in probiotic strains, treatment protocols, and outcome assessment.	[78]
Obesity	Meta-analysis of 20 RCTs (1411 participants)	*Lactobacillus gasseri* SBT2055; *Lactobacillus gasseri* BNR17; *Lacticaseibacillus casei* Shirota; *Lacticaseibacillus rhamnosus* CGMCC1.3724; *Bifidobacterium breve* B-3; representative multi-strain formulations	3–24 weeks	Probiotic supplementation did not significantly reduce body weight but significantly reduced BMI (MD −0.73 kg/m^2^; 95% CI −1.31 to −0.16), waist circumference, and hip circumference. The most promising effects were observed with selected *Lactobacillus* and *Bifidobacterium* strains.	Selected probiotic strains may serve as adjunctive therapy for overweight and obesity but should complement, rather than replace, lifestyle interventions.	Considerable heterogeneity in probiotic regimens, participant characteristics, and intervention duration; optimal strains and treatment protocols remain uncertain.	[79]
Metabolic syndrome	Meta-analysis of 11 RCTs (608 participants)	*Limosilactobacillus reuteri* V3401; *Lactobacillus acidophilus* La-5; *Bifidobacterium lactis* HN019; kefir-derived probiotic cultures; selected synbiotic formulations	8–12 weeks	Probiotic or synbiotic supplementation significantly improved BMI, LDL-C, and fasting blood glucose, whereas no significant effects were observed for most other cardiometabolic outcomes.	Probiotic or synbiotic supplementation may be considered as adjunctive therapy for improving selected cardiometabolic risk factors but should complement standard lifestyle management.	Small number of RCTs, heterogeneous probiotic/synbiotic formulations, and relatively short follow-up.	[80]
Psychobiotics (anxiety/depression)	Meta-analysis of 23 RCTs (20 included in quantitative synthesis; 1401 participants)	Representative *Lactobacillus* spp.; *Bifidobacterium* spp.; *Bacillus coagulans*; *Clostridium butyricum* MIYAIRI 588; representative single- and multi-strain formulations.	1–12 weeks	Probiotic supplementation significantly reduced depressive (SMD −0.96; 95% CI −1.31 to −0.61) and anxiety symptoms (SMD −0.59; 95% CI −0.98 to −0.19). Greater benefits were observed in clinically diagnosed patients receiving single-strain probiotics.	Selected probiotic strains may be considered as adjunctive therapy for patients with depression or anxiety but should not replace standard psychiatric treatment.	High heterogeneity in probiotic interventions, patient populations, and outcome measures; small sample sizes limited the certainty of evidence.	[81]

**Table 2:** Current knowledge gaps and future research priorities in probiotic therapy.

Current Challenge	Potential Impact	Future Research Priorities	References
Clinical and methodological heterogeneity	Limits comparison between studies	Standardized study design and outcome measures	[142,143]
Limited understanding of individual treatment response	Variable efficacy among patients	Identification of predictive microbiome and host biomarkers	[140]
Insufficient strain-specific evidence	Difficult extrapolation of results	Well-designed strain-specific randomized clinical trials	[20,144]
Variability in probiotic product quality	May influence safety and efficacy	Harmonized manufacturing standards and quality control	[15,26]
Limited long-term safety data in high-risk populations	Uncertainty regarding rare adverse events	Prospective safety studies	[145,146]
Differences in regulatory requirements	Variability in product evaluation and quality assessment across jurisdictions	International harmonization of regulatory frameworks	[26]

## Data Availability

No new data were created or analyzed in this study. Data sharing is not applicable to this article.

## Abbreviations

The following abbreviations are used in this manuscript:

RCT	randomized controlled trial
IBS	irritable bowel syndrome
IBD	inflammatory bowel disease
CDI	*Clostridioides difficile* infection
NEC	necrotizing enterocolitis
AAD	antibiotic-associated diarrhea
SCFAs	short-chain fatty acids
GALT	gut-associated lymphoid tissue
Treg	regulatory T cells
NF-κB	nuclear factor kappa B
MAPK	mitogen-activated protein kinase
WHO	World Health Organization
FAO	Food and Agriculture Organization
ESPGHAN	European Society for Paediatric Gastroenterology, Hepatology and Nutrition
AGA	American Gastroenterological Association
ISAPP	International Scientific Association of Probiotics and Prebiotics
